# Open MHC Class I Conformers: A Look through the Looking Glass

**DOI:** 10.3390/ijms22189738

**Published:** 2021-09-08

**Authors:** Fernando A. Arosa, André J. Esgalhado, Débora Reste-Ferreira, Elsa M. Cardoso

**Affiliations:** 1 Health Sciences Research Center (CICS-UBI), University of Beira Interior, 6200-506 Covilhã, Portugal; andre.esgalhado@fcsaude.ubi.pt (A.J.E.); deboraferreira@fcsaude.ubi.pt (D.R.-F.); cardoso.elsamaria@fcsaude.ubi.pt (E.M.C.); 2 Faculty of Health Sciences, University of Beira Interior, 6200-506 Covilhã, Portugal; 3 Health School, Guarda Polytechnic Institute, 6300-749 Guarda, Portugal

**Keywords:** HLA-ABC, open conformers, soluble HLA-I, cis-trans, associations, signaling, autoimmunity, cancer, transplantation, neuroimmunology

## Abstract

Studies carried out during the last few decades have consistently shown that cell surface MHC class I (MHC-I) molecules are endowed with functions unrelated with antigen presentation. These include cis–trans-interactions with inhibitory and activating KIR and LILR, and cis-interactions with receptors for hormones, growth factors, cytokines, and neurotransmitters. The mounting body of evidence indicates that these non-immunological MHC-I functions impact clinical and biomedical settings, including autoimmune responses, tumor escape, transplantation, and neuronal development. Notably, most of these functions appear to rely on the presence in hematopoietic and non-hematopoietic cells of heavy chains not associated with β2m and the peptide at the plasma membrane; these are known as open MHC-I conformers. Nowadays, open conformers are viewed as functional cis-trans structures capable of establishing physical associations with themselves, with other surface receptors, and being shed into the extracellular milieu. We review past and recent developments, strengthening the view that open conformers are multifunctional structures capable of fine-tuning cell signaling, growth, differentiation, and cell communication.

## 1. Introduction

**Figure 1:**
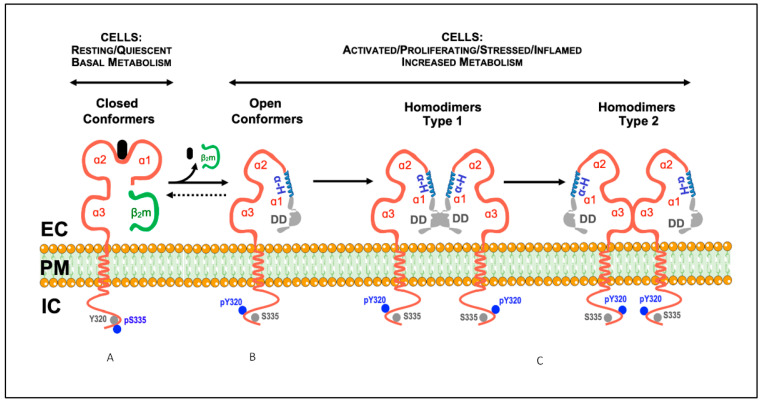
Model illustrating the described conformational states and cis-associations of cell surface MHC-I molecules. (**A**) Classical MHC-I molecules are trimeric composites of a transmembrane heavy chain (αHC) organized into three domains (α1, α2, and α3), non-covalently associated with a light chain (β2m) and a small peptide (**A**). These trimeric structures are differentially expressed at the plasma membrane of nucleated cells, and are also designated as closed conformers [5]. The cytoplasmic domain of the αHC of closed conformers contains two conserved motifs: (1) a tyrosine residue at position 320 in all HLA-A and HLA-B alleles that appears to be de-phosphorylated in resting cells (Tyr320, grey circles); (2) a serine residue at position 335 in all HLA-I alleles that appears to be phosphorylated in vivo (pSer335, blue circles). (**B**) Upon physiological settings associated with an increased metabolic activity (e.g., activation, proliferation, differentiation, etc.), a fraction of the closed conformers dissociate from the β2m and the peptide and generate free αHC, also known as open conformers. As a result, a physiological equilibrium exists where the closed/open conformers ratio decreases or increases depending on the metabolic state of the cell. Contrary to closed conformers, the cytoplasmic domain of the open conformers is serine de-phosphorylated (Ser335, grey circles) and tyrosine phosphorylated (pTyr320, blue circles). The phosphorylation status may allow membrane movement, localization and trafficking [5]. Thus, based on the current knowledge, pSer335 and pTyr320 may be considered as surrogate biomarkers of closed and open conformers, respectively (see [8,9,10,11,12,13,14,15], and text). (**C**) The open conformers formed at the plasma membrane of metabolically active cells may self cis-associate originating αHC homodimers, or hetero cis-associate originating αHC heterodimers (not shown, see Section 4). While some of these homodimers are non-covalently associated (see text), others are the result of the formation of disulfide bonds between unpaired cysteines located along the sequence of the αHC (see text). Depending on the orientation of the cis-association, two different homodimers may eventually form, type 1 and type 2. In this model, type 1 homodimers will preferentially be involved in trans-interactions with KIR and LILR receptors [5]. In contrast, type 2 homodimers, due to the flexibility of the α1 domain, namely the polymorphic and ordered α-helix (α-H, in blue), will favor cis-associations with nearby immune and non-immune receptors, such as CD8αβ and the insulin receptor (see text). DD, disordered domain (in grey); EC, extracellular milieu; PM, plasma membrane; IC, intracellular milieu.

Classical Major Histocompatibility Complex class I (MHC-I) molecules (HLA-A, HLA-B, and HLA-C in humans; H-2D, H-2K, and H-2L in mice) have a long past full of ins and outs and untold stories. They were initially identified as antigens involved in tissue rejection in mice and transfusion-related comorbidities in humans and, hence, called transplantation antigens [1]. Biochemical and molecular biology studies revealed that human and mouse classical MHC-I molecules present at the plasma membrane are trimeric structures formed by a heavy chain of about 45 kDa (thereafter, αHC), non-covalently associated with a 12 kDa beta2-microglobulin light chain (thereafter, β2m), and an 8–12 amino acid peptide. Seminal crystallographic studies revealed that the extracellular part of the αHC was organized into three domains: α1, α2, and α3. While the α3 domain is conserved, the α1 and α2 domains are highly polymorphic and form a groove where the peptide binds [2]. Before being expressed at the plasma membrane, the three components of the MHC-I molecules assemble in the endoplasmic reticulum (ER) through a series of complex processes that have been extensively studied [3]. In the ER, upon binding β2m and the peptide, the αHC folds into a closed/stabilized conformation [4]. Accordingly, the trimeric MHC-I molecules present at the cell surface are also known as closed MHC-I conformers [5]. Their primary function is immunological, namely to present peptides to CD8+ T cells and trans-interact with NK receptors [6,7]. Figure 1 illustrates the typical structure of a cell surface closed MHC-I conformer.

**Figure 2:**
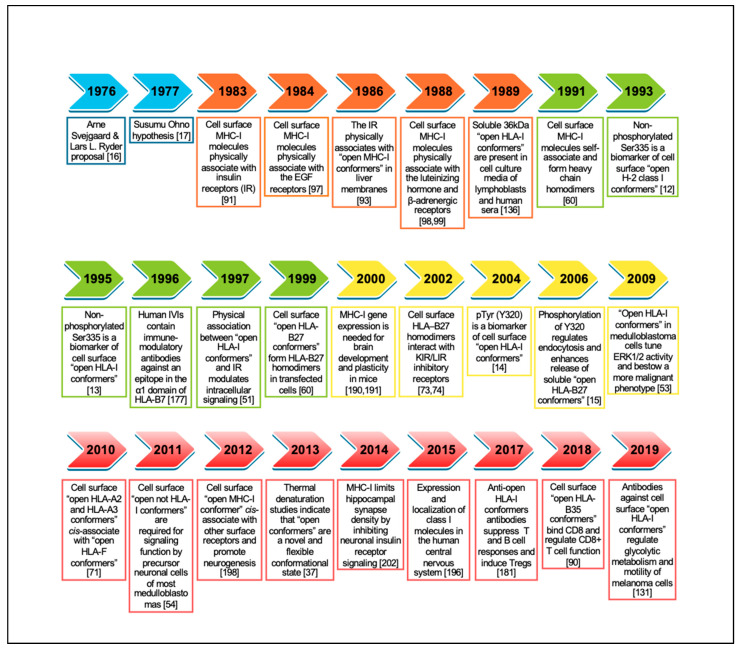
Timeline of representative studies contributing to the understanding of the biology of cell surface open MHC-I conformers. Initial hypotheses highlighted in blue. References for each hallmark are included between brackets.

Despite their prominent role in peptide presentation to CD8+ T cells, studies carried out during the 1960s–1970s noticed that MHC-I genes and molecules were associated with non-immunological functions. Thus, in the mid-1970s and 1980s, and as a result of experimental and clinical observations, farsighted scientists put forward four daring hypotheses. In 1976, Arne Svejgaard and Lars Ryder proposed that HLA class I molecules may interfere with ligand–receptor interactions not involved in immune reactions, explaining the reported associations between HLA-I alleles and metabolic and neurobehaviour disorders [16]. In 1977, Susumu Ohno suggested that the original role of cell surface MHC-I molecules was to serve as general anchorage sites of regulatory plasma membrane proteins involved in organogenesis and cell differentiation [17]. In 1985, Morten Simonsen and Lennart Olsson postulated that cell surface MHC-I molecules are a structural part of many receptors that they called compound receptors, with different biological functions [18]. In 1986, Michael Edidin summarized all these findings, including his own work with hormone receptors, and proposed that MHC-I molecules are likely part of functional hormone receptors and that their interactions modify the affinity of the receptors for their ligand [19]. Forty-five years later, these hypotheses have been confirmed to a greater or lesser extent, unveiling an uncharted territory hidden for a long time behind the looking glass. This review critically analyzes past and recent advances that shed light on the non-immunological functions of cell surface MHC-I molecules and their implications in biomedicine. Figure 2 presents a timeline of the most representative achievements that contributed to the body of knowledge on the biology of open MHC-I conformers.

## 2. Open MHC-I Conformers: Structure, Origin and the Search for a Physiological Function

### 2.1. Current Knowledge

Numerous studies have shown that under physiological settings related to an active cellular metabolism, the three constituents of the closed conformers present at the cell surface dissociate, leaving monomeric αHCs in the plasma membrane, nowadays known as open conformers [5]. These are mature and fully glycosylated proteins that hold an ordered and non-denatured structure. They are different from the immature, non-glycosylated open MHC-I conformers and their intermediates in the ER during assembly and transport to the cell surface [4]. The term “open conformers” highlights the fact that the dissociation of the peptide and the β2m from the αHCs leaves the α1 domain, but not the α2 and α3 domains, without the non-covalent forces that keep it folded when the peptide and the β2m are present. As a result, the α1 domain is free to unfold and adopt, what we call, an open conformation in the sense that its previous conformation was closed. Given its flexibility and mobility (see below), the α1 domain is also free to interact with any nearby matching terminal amino acid sequences and eventually affect the structural conformation of the α2 domain, which includes binding of exogenous peptides. The term “open conformers” does not include the non-peptide receptive forms described in other studies and discussed elsewhere [5]. Although being similar to other designations, such as peptide-empty, peptide-receptive, and β2m-free heavy chain, the term “open conformers” is comprehensive, and free of ambiguous or negative connotations. It refers only to αHCs that have dissociated from β2m and the peptide, as illustrated in Figure 1.

The identification of open MHC-I conformers at the cell surface of metabolically active cells was possible thanks to the development of monoclonal antibodies (mAbs) capable of recognizing conserved linear motifs in the α1 and α3 domains of the αHC when not associated with the β2m and the peptide (reviewed in [5]). In humans, open HLA-I conformers are present at the cell surface of activated T cells, B cells and monocytes, lymphoid cell lines, trophoblasts, and malignant cells, but not resting cells [5]. The presence of open MHC-I conformers observed in some blood cell samples reflects pre-existing primed/activated cells in peripheral blood, as is the case of ex vivo peripheral blood T cells and monocytes from patients with Ankylosing Spondylitis and Polycythemia Vera [20,21]. Open conformers are also present at the cell surface of β2m-deficient murine and human cell lines [22,23]. In this respect, studies performed in activated/transformed T cells suggested that cell surface open conformers were formed as a result of the endocytosis and intracellular trafficking of closed conformers through acidic vesicles. According to the authors, these conditions would favor the dissociation of the β2m and the peptide, leading to the formation of open conformers, which were then recycled back to the cell surface [24,25]. However, several subsequent studies have provided evidence that cell surface open conformers originate directly at the plasma membrane from closed MHC-I conformers after peptide and β2m dissociation [26,27] and that open conformers in H-2L^d^ transfected murine fibroblasts are endocytosed independently of closed conformers [28]. Furthermore, many studies have consistently documented that resting T cells do not express open MHC-I conformers at the cell surface [14,21,26,29,30], even though cell surface closed MHC-I conformers are constitutively endocytosed in resting T cells [31,32,33]. Therefore, although endocytosis and recycling of closed MHC-I conformers could contribute to the appearance of a fraction of open conformers at the cell surface, other mechanisms exist, which warrants further investigations.

The study of cell surface open MHC-I conformers had a significant boost by identifying conserved motifs in the cytoplasmic domain of the αHCs, which play essential roles in the biology of MHC-I molecules. The first motif contains a conserved tyrosine residue (Tyr320) within the amino acid sequence encoded by exon 6 of most mice and human αHC genes (except for HLA-C alleles) [34,35]. The second motif has two conserved serine residues (Ser332 and Ser335) within the amino acid sequence encoded by exon 7 of human and mice αHC genes, with Ser335 being constitutively phosphorylated (pSer335) in vivo [8,9]. Ensuing studies showed that pSer335 abrogated the binding of antibodies specific for a conserved sequence present in the cytoplasmic domain of H-2 class I molecules. These studies revealed that the antibodies specifically recognized cell surface H-2 class I molecules not associated with β2m, that is, open conformers [10,11,12]. Similar studies using human cell lines showed that the dissociation of β2m from cell surface HLA-I αHCs correlated with acquiring a cryptic epitope in the cytoplasmic domain, likely resulting from de-phosphorylation of Ser335 [13]. These results indicated that MHC-I molecules having pSer335 were in the closed conformation and that Ser335 de-phosphorylation was associated with the open conformation. Years later, studies with normal human peripheral blood T cells activated in vitro revealed that cell surface open HLA-I conformers, but not closed ones, were tyrosine phosphorylated, most likely at Tyr320 [14]. Interestingly, the substitution of Tyr by Phe abrogated endocytosis of HLA-B27 molecules, pointing to this Tyr residue as critical for endocytosis [15]. Altogether, these studies revealed that the phosphorylation status of the cytoplasmic Tyr320 and Ser335 residues in cell surface MHC-I molecules was a biochemical feature that distinguished between cell surface closed conformers (pSer335) and cell surface open conformers (pTyr320), and was perhaps involved in membrane movement and intracellular trafficking [5].

It is now known that several physiological settings induce the formation of open MHC-I conformers at the plasma membrane. These include cell activation and proliferation, cell growth and differentiation, and inflammation. As discussed elsewhere [5], these findings led some authors to propose the existence of a physiological equilibrium between open and closed conformers at the plasma membrane of cells, which the metabolic state and nutritional needs will influence. In this regard, biochemical and cell biology studies analyzing the kinetics of expression of cell surface closed and open HLA-I conformers in normal human T cells activated in vitro unveiled essential aspects of the biology of MHC-I molecules [14]. First, a shift from the closed to the open forms during the proliferative phase of the T cells. Second, the physical cis-association of cell surface open HLA-I conformers with CD8αβ–Lck complexes pointing to this kinase as a likely candidate for the phosphorylation of Tyr320 observed in the open conformers. Third, the existence of a shift from the open to the closed conformers during the cessation of T cell proliferation leading the closed and open HLA-I conformers to return to baseline levels.

Despite this knowledge, cell surface open MHC-I conformers were considered unstable structures that shared similarities with a molten globule state [36]. However, recent studies using molecular dynamics simulations and thermal denaturation measured by tryptophan fluorescence have revealed that not all open conformers are molten globules [37]. Indeed, most open HLA-I conformers are pretty stable and display high molecular flexibility [38,39]. Notably, the α1 domain of the open conformers appears to be in a relatively extended conformation, which allows lateral cis-interactions with nearby receptors [40,41,42]. Moreover, studies using novel NMR techniques, such as heteronuclear single quantum correlation spectroscopy and chemically induced dynamic nuclear polarization, have revealed that a molten globule protein is flexible and has functional significance under physiological conditions [43]. Relevant to this review is the fact that the functional specificity of molten globule proteins relies on establishing cis-interactions with functional ligands [44,45]. In this context, two crystallographic studies of mouse and human open conformers have provided insightful data. The first study revealed a sequence in the α1 domain of the murine H-2L^d^ class I αHC (encompassing residues 46–53) containing a tryptophan at position 51. It showed that Trp51 rotates away from the rest of the αHC in the open conformation, becoming recognizable by the 64-3-7 antibody specific for open H-2L^d^ conformers [46]. In the second study, the authors designed a disulfide-stabilized version of the human class I molecule HLA-A2 without a peptide. Using X-ray crystallography and molecular dynamics simulations, the authors showed the exchange of cognate peptides. They also revealed that the amino acid side chains lining the binding pockets switch between a peptide-free open conformer and a peptide-bound closed conformer [47]. These studies are, perhaps, the first molecular evidence that the open conformers may interact with nearby matching peptide sequences of cell surface receptors, as shown for the N-terminal amino acid sequences of CD8α and CD8β [48].

### 2.2. Forward-Looking Perspective

In summary, cell surface open MHC-I conformers can be viewed as novel structures expressed by metabolically active cells over periods of time, in the course of which they may fine-tune critical immunological and non-immunological physiological processes [5]. The latter is of foremost importance during specific periods of the lifetime of a cell; for instance, when normal human T cells receive antigen-dependent or antigen-independent signals to enter the cell cycle, grow, proliferate and differentiate [14,49,50], or when tumor cells receive signals from hormones and growth factors to fine-tune intracellular signaling [51,52,53,54]. The evidence accumulated over the years suggests that the physiological functions of the open MHC-I conformers expressed at the plasma membrane of metabolically active cells ultimately depend on the fate of the αHCs.

**Figure 3:**
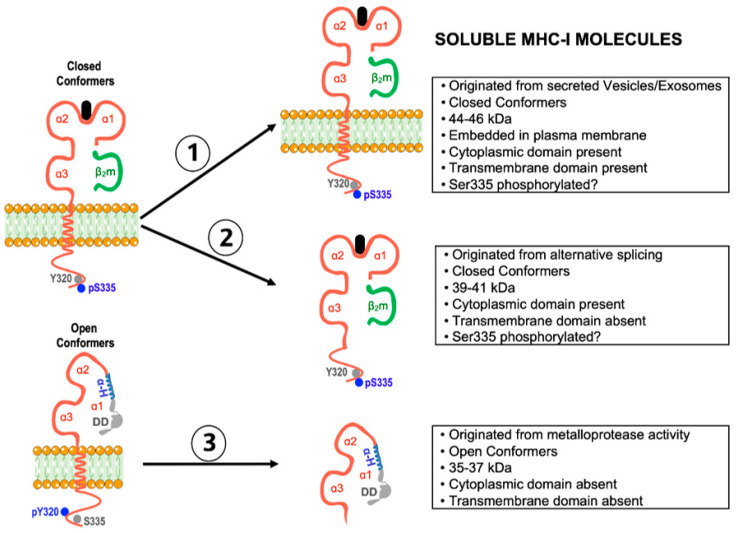
Schematic representation of the three main forms of soluble MHC-I molecules and their features. Soluble form 2 is not thought to be present at physiologically relevant levels in serum, leaving soluble forms 1 (closed conformers) and 3 (open conformers) as the main soluble forms (see text for more details).

Hitherto, three major fates can be envisaged for the αHCs. First, to self cis-associate and form αHC homodimers (Figure 1). These are de novo cell surface receptors able to trans-interact with inhibitory and activating receptors, suppressing or boosting immune responses. Cell surface homodimers have essential implications for (auto)immune responses. Second, to cis-associate with other surface receptors to form αHC heterodimers. These are de novo cell surface compounds, whose formation allows the αHCs to fine-tune receptor-mediated signaling. Of note, cell surface αHC heterodimers may impact cancer evasion and neuronal function (see below). Third, to be shed from the plasma membrane or after internalization as soluble open αHC conformers (Figure 3). Soluble αHCs are de novo circulating molecules capable of trans-interacting with other receptors and inducing the formation of specific antibodies, with implications for graft rejection and neuronal growth. Although endocytosis and recycling of plasma membrane open conformers may also be envisaged as an additional fate resulting in antigen cross-presentation, this evidence is limited to transfected transformed cells [55,56]. In this regard, recent studies have shown that open conformers are either degraded in the lysosomal compartment or secreted in a soluble form via secreted exosomes [57,58], as shown for the closed conformers [59], thus contributing to the pool of open sHLA-I conformers. In the following sections, we will elaborate further on these three major fates in light of past and recent developments. We will finalize this review by analyzing recent data on the possible role played by the open conformers in the central nervous system.

## 3. Homo-Associations of Open MHC-I Conformers: Modulation of (Auto)Immune Responses

### 3.1. Current Knowledge

The existence of cis-associations between open conformers (i.e., αHC homodimers) was first described by flow cytometric energy transfer in human B-lymphoblastoid cells and HLA-A2-carrying liposomes [60]. Then, immunoprecipitation studies showed that the αHC homodimers found in H-2L^d^, H-2D^b^, and H-2D^d^ transfectants were disulfide-linked via a conserved cytoplasmic cysteine [61]. It is worth noting that studies using advanced fluorescence techniques showed that in activated normal lymphocytes, lymphoblastoid cell lines, and transformed fibroblasts, the presence of surface open HLA-I conformers correlated with the formation of HLA-I clusters. These clusters were made of closed conformers, open conformers, and growth factor receptors, such as IL-2R and IL-15R [62,63,64]. In these studies, no HLA-I clusters were observed in the surface of resting T or B cells or normal fibroblasts, which agrees with reports showing that cell surface open HLA-I conformers are absent or expressed at baseline levels by normal resting T cells [14,21,26,29,30]. Notably, HLA-I clustering was reversed by adding exogenous β2m, suggesting that cell surface open HLA-I conformers were non-covalently associated and in physiological equilibrium with closed conformers [62,63]. This assumption is supported by studies showing that non-covalent MHC-I cis-associations at the cell surface occur only between open MHC-I conformers after dissociation of β2m and the peptide from the αHC [42]. Noteworthy studies examining the expression and half-life of cell surface open HLA-B27 conformers showed that the addition of exogenous peptide ligands reduced the number of open HLA-B27 conformers, reinforcing the view that surface open HLA-I conformers originate primarily from closed HLA-I conformers following β2m and peptide dissociation [27].

Soon afterward, immunoprecipitation studies in HLA-B27 transfected cells reported that cell surface open HLA-B27 conformers cis-associated and formed HLA-B27 dimers. These associations relied on the formation of disulfide bonds between unpaired cysteine residues at position 67 (Cys67) [65], corroborating earlier findings with H-2 class I transfectants [61]. Then, biochemical studies in human cells showed that cell surface cis-associations between open HLA-I conformers, involving disulfide bonds, also exist at the cell surface of normal peripheral blood human T cells activated in vitro [14] and trophoblasts [66]. It is presently known that other human HLA-I molecules share the capacity of HLA-B27 molecules to cis-associate via disulfide bonds. Thus, the formation of αHC homodimers also occurs with classical HLA-A, HLA-B, and HLA-C [67,68], non-classical HLA-G and HLA-F [69,70], and the glycolipid-binding molecule CD1d [71], via covalent (disulfide bonds) and non-covalent cis-associations. Subsequent studies with the HLA-B27 allele illustrated the physiological significance of the presence of open conformers at the cell surface, by showing that cell surface open HLA-B27 conformers, expressed either as single αHCs or as disulfide-linked αHC homodimers, were novel ligands of Killer cell Ig-like Receptors (e.g., KIR3DL2) and Leukocyte Ig-like Receptors (e.g., LILRB1 and LILRB2) present on lymphomyeloid cells, thus modulating the immune response [72,73]. Subsequent studies showed that other HLA-I alleles also share this trans-interaction property. Indeed, open HLA-G conformers are recognized by LILRB1 [74], while HLA-G dimers exhibit a high affinity for LILRB1 and LILRB2, also known as ILT2 and ILT4, respectively. Notably, the interaction between HLA-G dimers with LILRB1 enhanced the ILT2-mediated signaling at the cellular level [75]. On the other hand, cell surface open HLA-F conformers cis-associate with open HLA-A2 and HLA-A3 conformers, but not with closed ones [70]. Subsequent studies identified NK receptors KIR3DL2, KIR2DS4, and KIR3DS1 as novel ligands of the open HLA-F conformers and of a variety of classical open HLA-I conformers, such as HLA-A2, HLA-A3, and HLA-B7 [76,77,78].

In this context, it is essential to mention that disulfide bonds between cytoplasmic unpaired cysteines, namely C308, C320, C326, and C339, are required to form cell surface αHC homodimers and the trans-interaction with KIR and LILR receptors [67,68]. Furthermore, some of these studies showed that HLA-F and HLA-I open conformers cooperated on transformed lymphoid and myeloid cell lines to cross-present exogenous antigens after endocytosis and trafficking through endolysosomes [56]. These results are reminiscent of earlier studies in dendritic cells transfected with wild-type H-2K^b^ molecules or H-2K^b^ molecules lacking exon 6 or having a Tyr to Phe substitution [35]. These investigations showed that wild-type H-2K^b^ molecules were endocytosed and routed to endolysosomal compartments where they were capable of binding peptides originated from extracellular antigens, thus facilitating cross-presentation and boosting CD8+ T cytotoxic responses. Overall, these data indicate that the routing of mice and human MHC-I molecules to endolysosomal compartments relies on the phosphorylation of Tyr320, a biochemical process that appears to occur in open conformers [14,15].

### 3.2. Forward-Looking Perspective

**Figure 4:**
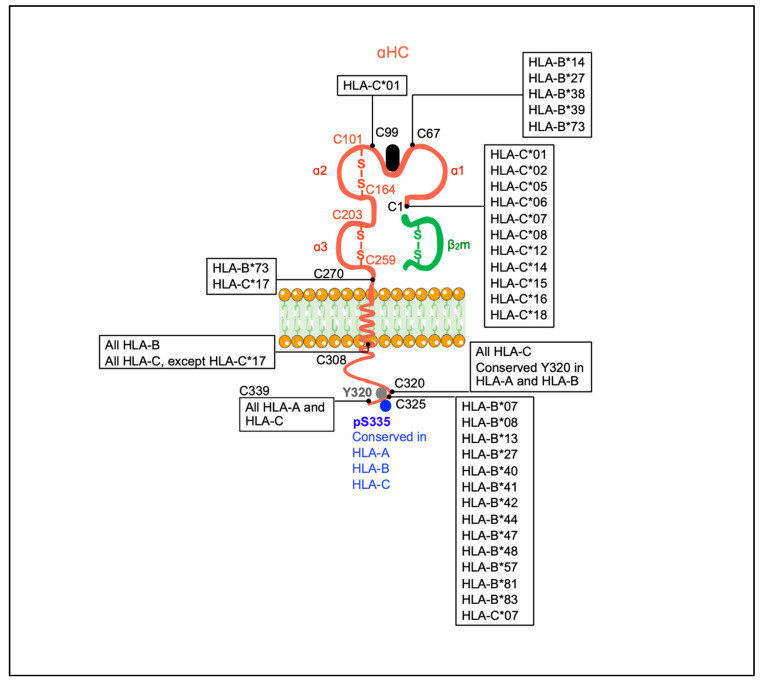
Summary of unpaired cysteines in the αHC of classical human HLA-I molecules. Paired cysteines involved in disulfide bonds in the α2 and α3 domains are shown in red. Unpaired cysteines are identified in black and are the following: C1, C67, C99, C270, C308, C320, C325, and C339. Human HLA-I alleles having each of the referred unpaired cysteines in their sequence are indicated. *, separator between the gene and allele group (http://hla.alleles.org/nomenclature/naming.html). Accessed on 7 September 2021. All information retrieved from IPD-IMGT/HLA, Release 3.40.0, 2020-04-20 (https://www.ebi.ac.uk/ipd/mhc/alignment). Accessed on 31 May 2021. Robinson, J, Barker, DJ, Georgiou, X, Cooper, MA, Flicek, P, Marsh, SGE. The IPD-IMGT/HLA Database. Nucleic Acids Research (2020) 43:D948-D955.

Some previous reports have shown the expression of open HLA-I conformers at the cell surface of ex vivo peripheral blood T cells and monocytes in patients with chronic inflammatory disorders [20,21], pointing to systemic inflammation as an inducer of the shift from the closed to the open forms. Thus, chronic inflammation is characterized by high levels of IFN-β, IFN-γ, and TNF-α [79,80]. These cytokines use signaling pathways that enhance MHC-I gene transcription and induce an increase in the expression of closed and open MHC-I conformers at the plasma membrane of a variety of cell types, including tumor cells [81,82,83,84]. Therefore, within an inflammatory context, such as during malignant transformation, the expression of open conformers and αHC homodimers is a likely outcome. In line with the studies mentioned above, the likelihood that open conformers and αHC homodimers expressed by tumoral cells trans-interact with inhibitory or activating receptors present on effector T and NK cells, downplaying or boosting antitumor responses, as shown for HLA-G dimers [85], is a possibility that deserves further investigations. In this respect, it is worth mentioning studies showing that a shift from the closed to the open conformers in target cell lines confers protection against T and NK cell recognition and killing [86,87]. Together with the number of unpaired cysteines observed among different HLA-I alleles (Figure 4), these facts anticipate insightful results in clinical settings where deregulated immune responses may determine the development and progression of chronic inflammatory diseases. Indeed, it is presently avowed that open conformers, αHC homodimers, and HLA-I clusters are plasma membrane structures that modulate innate and adaptive responses [72,73,88,89]. Therefore, even though the physiological function of αHC homodimers expressed by tumoral and non-tumoral cells is primarily immunological, the possibility that they may also play non-immunological functions cannot be ruled out. However, as discussed in the next section, the open conformers may also cis-associate with other receptors and fine-tune activation and survival signals, thus playing a non-immunological function.

## 4. Hetero-Associations of Open MHC-I Conformers: Modulation of Cell Signaling and (Tumor) Cell Growth

### 4.1. Current Knowledge

The description of hetero-associations between MHC-I molecules and other cell surface receptors (i.e., αHC heterodimers) preceded the characterization of cell surface αHC homodimers. Initial studies showed that the H-2 class I complex gene products interfered with the binding of glucagon and insulin to their receptors in liver plasma membranes. They also showed that different murine H-2 class I haplotypes were associated with differences in the stimulation of adenylate cyclase and the generation of cAMP in response to glucagon or insulin treatment (reviewed in [19]). Thus, liver and spleen cells from animals with the H-2^b^ haplotype produced higher levels of cAMP (about 50% higher) than animals with the H-2^k^, irrespective of the mouse strain [19].

The existence of cis-interactions between HLA-I molecules and hormone receptors at the plasma membrane was supported and extended by immunoprecipitation studies, showing that a fraction of insulin receptors were physically associated with MHC-I molecules in a variety of human cell types [90,91,92,93,94]. Interestingly, as in mouse, the HLA-I alleles influenced insulin binding [95]. Thus, by using human B lymphoblasts transfected with different HLA-I alleles, it was shown that cell lines expressing HLA-B5, either alone or together with other HLA-I alleles (e.g., A1, A2, B8, or C alleles), bound insulin with moderate to low affinity and appeared to carry a single type of binding site. In contrast, the cell lines lacking HLA-B5 and being positive for other HLA-A or HLA-B alleles bound insulin with higher affinities (5–6 fold higher) and appeared to carry two types of insulin binding sites [95].

Besides the insulin receptor (IR), MHC-I molecules have also been shown to cis-associate with the epidermal growth receptor (EGFr) [96], the luteinizing hormone receptor [97,98], the β-adrenergic receptor [98,99], the ɣ-endorphin receptor [100], the IL-2 and IL-15 receptors [101,102,103], and the transferrin receptor [104]. Cis-associations between HLA-I molecules and receptors have not been restricted to hormone, cytokine, and growth factor receptors. Thus, several surface immunoreceptors have been found to cis-associate with MHC-I molecules, including CD8αβ [14,105,106], CD82 [107], and a variety of members of the NK receptor family, including KIR, LILR, and CD94-NKG2 [108]. The cis-associations between cell surface MHC-I molecules and NK receptors have been reported in NK cells, mast cells, and osteoclasts. They have functional implications on the cells, including modulation of NK cytotoxicity, mast cell activation, and osteoclast development [109,110,111] (see Table 1).

Unlike the cis-associations with NK receptors, which appear to occur with closed MHC-I conformers [108], most studies point out the pool of open MHC-I conformers as the direct partner of the receptors. Thus, immunoprecipitation studies using antibodies against ^125^I-labeled photoreactive insulin bound to the IR or against the insulin binding site showed co-immunoprecipitation of the MHC-I αHC, but not of the β2m light chain, clearly indicating that the IR was physically associated with open MHC-I conformers [92,93]. Similar results were obtained in studies examining conformational changes of the IR after insulin binding, i.e., co-immunoprecipitation of the αHC and not β2m [114]. Notably, most of these studies provided strong evidence that open MHC-I conformers present at the cell surface were involved in the modulation of IR-signaling and endocytosis after insulin binding, two biochemical processes likely resulting from conformational changes in the structure of the IR after insulin binding [115]. Insulin binding is known to induce conformational changes in both the extracellular and intracellular domains of the IR α and β subunits [116,117,118,119]. Of note, the conformational change in the extracellular part of the IR exposes an otherwise cryptic terminal sequence [120,121].

Ensuing studies with the IR demonstrated that the “open αHC:IR:insulin” complex readily exists at the plasma membrane of hepatocytes and adipocytes and plays an important biological role in IR function [51]. By using B lymphoblastoid cell lines, the investigators unveiled important physiological aspects of the interaction between HLA-I molecules and the IR. First, the formation of the HLA-I:IR complex relied on the presence of open HLA-I conformers, something already expected. Second, the affinity of insulin for the IR and the IR-associated tyrosine kinase activity augmented as the HLA-I:IR ratio increased. Third, an increase in the HLA-I:IR ratio enhanced phosphorylation of the IR substrate-1 (IRS-1) and the activation of phosphoinositide 3-kinase (PI3K). Last but not least, all these effects were reduced or abrogated if the cells were incubated with exogenous β2m, which induces dissociation of the “open αHC:IR:insulin” heterodimers [51]. The non-immunological function of open HLA-I conformers present at the cell surface, through their physical cis-association with growth factor receptors, namely the IR, was recently supported and extended by a series of studies conducted in human medulloblastoma cell lines characterized by low expression of closed HLA-I conformers, a feature associated with a more malignant phenotype and a poorer prognosis. These studies provided molecular evidence for the involvement of cell surface open HLA-I conformers in the activation of ERK1/2 and AKT kinases, two downstream signaling molecules used by many growth factor receptors [53,54]. The authors concluded that fine-tuning of intracellular signaling in medulloblastoma and other cancer cells could be achieved through cis-associations between open HLA-I conformers and growth factor receptors upon ligand binding. These findings are evocative of earlier studies with murine B16BL6 melanoma cell clones expressing low closed H-2K conformers. In these malignant cells, fundamental biological processes mediated by activation of AKT, such as proliferation and resistance to apoptosis induced by deprivation of serum-derived growth factors, were increased and allowed the cells to grow and induce tumors when transfused into mice [52,122]. In this regard, it has been consistently shown that the expression of open MHC-I conformers often parallels the low expression of closed MHC-I conformers by tumor cells. This combination is a distinguishing feature of cancerous cells with prolonged survival [83,84,123,124,125,126]. The fact that expression of open MHC-I conformers on the cell membrane of cancerous cells does not confer protection from cytotoxic CD8+ T cells and NK cells [86,87] reinforces the view of a non-immunological function of cell surface open MHC-I conformers.

Despite their paramount importance, these studies left a fundamental question unanswered. Which part/motif of the open αHC conformer physically interacts with the receptors? Crystallographic studies of open conformers complexed with hormone or growth factor receptors are lacking and would undoubtedly help unveil this vital issue. Meanwhile, a series of dazzling studies in the 1990s provided indirect but strong evidence for the involvement of the α1 domain of the αHC of MHC-I molecules in the hetero cis-associations with hormone receptors. Initial studies showed inhibition of IR internalization by a 25-amino-acid peptide derived from the α1 domain of H-2Dk molecules [127,128]. As a result, the number of IR at the plasma membrane of adipocytes increased, and the effect of insulin lasted longer. Similar results were reported with the insulin-like growth factor receptors IGF-I and IGF-II [129]. Interestingly, these studies identified a target sequence in the N-terminal domain of the IR α-subunit, having amino acid similarities to the bioactive peptides [130]. These studies showed that the bioactive peptides did not bind to the peptide-binding groove. Rather, they bound to the α1 domain, thus indicating that their inhibitory effect on IR internalization was a direct consequence of this binding. According to the authors, the immediate outcome was to hamper the normal biological function of the flexible α1 domain of the open conformer [130]. These investigators named the α1 domain the “master-key”, a flexible polypeptide with a peptide sequence whose function would bind to cryptic motifs present in various hormone and growth factor receptors, including the IR, EGFr, IGF-Ir, and IGF-IIr. The cryptic motifs are revealed only after binding the physiological ligands to their receptors. The physical cis-interaction between the α1 domain and the cryptic sequence regulates signaling and facilitates endocytosis [131]. As discussed later, recent investigations with neuronal and glial cells have revived these studies by showing that the crucial roles played by MHC-I molecules in brain function are closely linked to neuronal insulin receptor expression and function.

### 4.2. Forward-Looking Perspective

**Figure 5:**
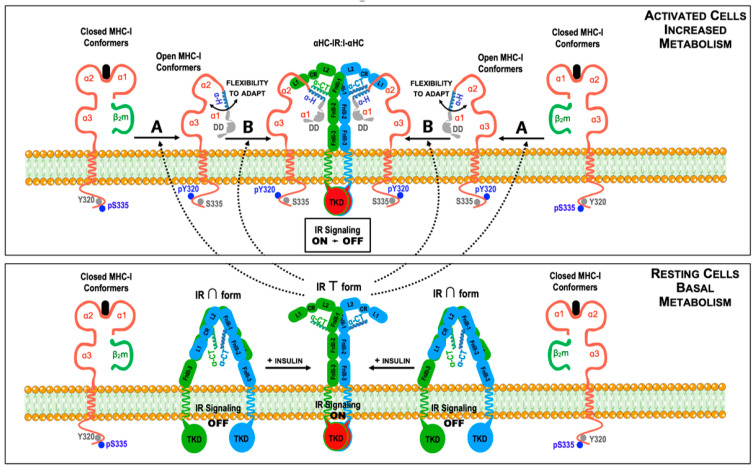
Model for the regulation of insulin signaling by cis-association between cell surface MHC-I molecules and insulin receptors (IR) in insulin-dependent tissues. In resting cells (lower figure), the large majority of MHC-I molecules expressed at the cell surface are closed conformers. Likewise, under resting conditions and low circulating insulin levels, the IR is in its inactive ∩ conformation (signaling off), with the α-C-terminal domains (α-CT) of the IRα chains hidden [120,121]. An increase in the metabolic activity of the cells and the presence of insulin in the external milieu will result in binding of insulin to the IR, inducing a shift towards the active T conformation (signaling on) and the exposure of the α-CT domains [116,117,118,119,120,121]. Under these conditions, the T conformation of the IR with the bound insulin may follow two paths (upper figure). A, to induce the dissociation of β2m and the peptide from nearby closed MHC-I conformers, generating open conformers. B, to directly associate with pre-existing open conformers. In either case, the cis-association may occur between the α-CT domains of the α extracellular chains of the IR and the α-helix (α-H) of the α1 domains, leading to the formation of a complex constituted by one IR:I complex and two αHCs (αHC-IR:I-αHC). Alternatively, the α-helix (α-H) of the MHC-I α1 domains may also interact with N-terminal sequences of the transmembrane β chains of the IR (not shown). Phosphorylated amino acid residues in the cytoplasmic domain of MHC-I molecules are indicated, as in Figure 1. Whether the Tyrosine Kinase Domains (TKD) of the β chains of the IR are involved in the phosphorylation of Tyr320 is not known, but is a likely possibility.

Overall, it can be concluded that the αHC homodimers have mainly physiological functions related to the regulation of immune responses. These functions occur at a systemic level and are easily measurable. In marked contrast, αHC heterodimers have primarily non-immunological functions related to the physiology of the cell. These functions occur at the plasma membrane of metabolically active cells, and the outcomes are more challenging to measure. However, they are no less important. Thus, the studies mentioned above have provided robust data to draw a picture where the binding of hormones and growth factors to their receptors induces conformational changes that allow physiologically relevant cis-associations with nearby MHC-I conformers. Using the insulin receptor (IR) as a prototype, and based on the current knowledge, a model can be proposed where the conformational change that occurs in the extracellular part of the IR after insulin binding exposes a cryptic terminal amino acid sequence that may act, at least, in two ways. First, it may compete with the bound peptide from near closed MHC-I conformers, inducing the sterical dissociation of β2m [92]. Second, it may bind to nearby open MHC-I conformers through matching sequences in the α1 domain, functioning as a cognate peptide [5]. In either case, a physical cis-association between open MHC-I conformers and the IR would take place and allow the αHC to fine-tune IR signaling and function (Figure 5). Although this model does not contemplate a possible role of soluble open MHC-I conformers on IR-mediated signaling, it may be envisaged that they could compete with the binding of the plasma membrane open conformers via the same motifs. In any case, these views may be incorporated, and the model improved when more data on the molecular and cell biology aspects of αHC heterodimers and soluble open MHC-I conformers become available. In any case, this model can be used to predict physiological outcomes of the cis-association between cell surface open MHC-I conformers and the IR, among other hormone and growth factor receptors (see Box 1). In this respect, and in agreement with some of the above-referenced reports, it is worth mentioning a recent study showing that antibodies against cell surface open HLA-I conformers interfere with the glycolytic metabolism and motility in melanoma cells [132], reinforcing once more the non-immunological functions played by the open MHC-I conformers.

Box 1 **Physiological implications for the cis-associations between open MHC-I conformers and insulin receptors.**

In the model depicted in Figure 5, at least three different scenarios can be envisaged depending on the amount of MHC-I molecules at the plasma membrane.

1. The cells express baseline levels of MHC-I molecules and the open MHC-I conformers will homeostatically regulate IR-mediated signaling and glucose uptake as needed.
2. The cells show an increased expression of MHC-I molecules at the plasma membrane (above baseline levels, for example, caused by inflammatory cytokines). In this setting, many αHC-IR:I-αHC complexes will be formed at the cell surface with a subsequent increase in IR-mediated signaling. As a result, extracellular glucose levels may temporarily decrease, leading to hypoglycemia.
3. The cells show a diminished expression of MHC-I molecules at the plasma membrane (below baseline levels, for example, caused by genetic or microenvironmental factors). In this setting, very few αHC-IR:I-αHC complexes will form and the number of IR present at the plasma membrane will increase due to a reduced endocytosis. As a result, IR-mediated signaling will be constitutively turned on. Although, initially, extracellular glucose levels may decrease, the cells will become refractory to insulin, which may ultimately lead to hyperglycemia and type II diabetes.

This model is based on the following evidence:

- Studies with peptides derived from the α-helix of the α1 domain, which inhibit the formation of αHC-IR:I-αHC complexes in adipocytes [92,127,128,129,130,131].
- Studies showing that the IR cis-associates with open αHC conformers [51,91,92,93].
- Studies showing that insulin binding to the IR induces a conformational change that facilitates cis-association with open MHC-I conformers [114,115,130].
- Studies with neuronal cells showing that IR-mediated signals regulate synapse number and plasticity, and that changes in the expression levels of MHC-I molecules interfere with IR-mediated signaling [133,134].

The model is testable and may be influenced by many factors, including, among others:

- Whether uptake of glucose by the cells is dependent or independent of insulin signaling;
- Whether the cells are part of a tissue/organ or are circulating lymphomyeloid cells;
- Whether they are normal or malignant cells.

## 5. Shedding of Open MHC-I Conformers: Modulation of Tumor Cell Growth and Allograft Rejection

### 5.1. Current Knowledge

Soluble HLA-A2 and HLA-B7 molecules (in the following, sHLA) in sera of healthy individuals have been known about since the early 1970s [135,136]. Following this, studies showed that sHLA-I molecules were biochemically complex and contained different molecular forms [137,138,139,140]. At present, sHLA-I molecules comprise three main pools that differ in molecular mass and domain composition ([141], Figure 3). The first pool contains sHLA-I conformers of about 44–46 kDa with transmembrane and cytoplasmic domains, and β2m, representing plasma membrane-embedded closed HLA-I conformers, is most likely present in small extracellular vesicles and exosomes [59,137]. The second pool contains sHLA-I conformers of about 39–41 kDa, lacking the transmembrane domain, resulting from the removal of exon four by alternative splicing [138,139]. The third pool contains smaller αHCs with a 35–37 kDa molecular mass, not associated with β2m, and lacking transmembrane and cytoplasmic domains [140]. In human cells, these smaller sHLA-I conformers are derived from cell surface open conformers after proteolytic cleavage by a Zn-dependent membrane metalloprotease [142]. This pathway also releases soluble open conformers of non-classical HLA-E, HLA-G, and CD1d molecules [143].

Quantitative studies using conformation-specific monoclonal antibodies concluded that the levels of closed and open sHLA-I conformers in sera of healthy people were roughly 1.50 μg/mL and 0.25 μg/mL, respectively [144,145,146,147]. These studies also showed that the levels of closed and open sHLA-I conformers were unrelated, suggesting different release mechanisms. However, the serum levels were influenced by the HLA-I alleles. Individuals that were A9 (now A24), A23, A28 (now A68), A29, Aw33, B7, B13, Bw65 (now B14), or Cw8 showed mean serum concentrations of closed sHLA-I conformers higher than individuals with other alleles, with individuals having A23, A24, B14 and Cw8 showing the highest serum levels. Of note, individuals that were B35 and Cw4 secreted about 10-fold more open sHLA-I conformers than individuals that were B17, suggesting that HLA-B35 and HLA-Cw4 are prone to become open conformers followed by cleavage and shedding. Interestingly, these data agree with recent studies showing that HLA-B35 molecules are expressed as open conformers at the cell surface of antigen presenting cell lines [89]. Moreover, the influence of the HLA-I alleles on the levels of sHLA-I conformers agrees with reports mentioned above, showing that HLA-I alleles influence the binding of hormones to their receptors and the formation of αHC homodimers [88,95].

Soluble HLA-I conformers were initially considered molecules with no biological function, as with cell surface open conformers. However, the accumulated body of evidence from experimental and clinical investigations indicates that sHLA-I conformers, both closed and open, are endowed with immunoregulatory properties [148,149,150]. This role would be more relevant in settings where sHLA-I conformers are increased, including viral infections [151,152], autoimmune disorders [153,154,155], hematological and solid tumors [156,157,158,159], after transplantation [160,161], after blood transfusion [162,163], and during aging [164]. The increase in serum sHLA-I levels is associated with cellular activation and high levels of cytokines such as IFN-γ. Indeed, the upregulation of cell surface expression of closed HLA-I conformers induced by IFN-γ has as an immediate outcome: the induction of a shift from the closed conformers to the open conformers, favoring their subsequent release into the extracellular milieu [165,166,167,168]. The proposed mechanisms of immunoregulation used by sHLA-I conformers include the induction of apoptosis of alloreactive CD8+ T cells, virus- and tumor-specific CD8+ T cells, and NK cells. The inhibitory and cytotoxic effects of sHLA-I conformers appear to be mediated by the binding of the α3 domain of sHLA-I conformers to the CD8α chain, and KIR expressed by NK and CD8+ T cells [169,170,171]. The molecular mechanisms of apoptosis induction or cytotoxicity inhibition are linked to the secretion of CD95L and TGF-β1 [172,173,174]. Due to their capacity to inhibit cytotoxic activities, sHLA-I conformers are considered tolerogenic factors associated with tumor escape and the immunosuppressive effects of blood transfusions and apheresis [175,176,177,178]. Interestingly, in parallel with the studies with sHLA-I molecules, functional studies with peptides derived from the α1/α2 domains of HLA-I molecules showed that they also induced immunotolerance [179].

The existence of sHLA-I conformers in various body fluids raised the question of whether such structures would elicit the formation of antibodies. The answer came from studies carried out with intravenous immunoglobulins (IVIs) obtained from large plasma pools of healthy donors. These studies revealed the existence in human serum of anti-HLA-I antibodies recognizing a conserved peptide sequence in the α1 domain of the HLA-B7 molecule, which is present in other HLA-I alleles [180]. Of note, this study showed that the anti-HLA-I antibodies had, like sHLA-I conformers, inhibitory activity against virus-specific and allogeneic cytotoxic CD8+ T cells. Presently, therapeutic IVIs are widely used to treat many autoimmune diseases and prevent infections and graft-versus-host reactions because they have anti-inflammatory and immunomodulating properties [181]. These properties rely on recognizing soluble and membrane-associated immunological molecules, including HLA-I molecules [182]. More recently, a series of studies revealed that human IVIs contain antibodies against open HLA-I conformers. Importantly, these antibodies are also endowed with immunosuppressive properties towards antibody-producing plasma B cells and activated CD4+ T cells, perhaps through the induction of regulatory T cells [183,184,185].

These results are most relevant in the context of transplantation. Thus, the presence of preformed antibodies against donor HLA-I molecules in the graft recipient, and known as donor-specific antibodies (DSA), can elicit a series of pathologic features after interaction with closed HLA-I conformers expressed in the graft vasculature. Triggering of inflammatory pathways induced by DSA in endothelial cells occurs through physical cis-associations with endothelial receptors, such as the β4 integrin, leading to the activation of kinases ERK and AKT [113]. However, several reports have documented that DSA contain a mixture of antibodies against closed and open HLA-I conformers, which may influence the extent of the damage. Thus, while DSA against closed HLA-I conformers are pathogenic and trigger inflammatory pathways that lead to graft rejection, DSA against open HLA-I conformers are not pathogenic [186,187], most likely because of their capacity to induce immunotolerance [183,184,185].

### 5.2. Forward-Looking Perspective

The results summarized above are evocative of earlier in vitro studies showing that soluble antibodies against cell surface closed MHC-I conformers influenced signaling pathways in immune and non-immune cells ([188], and references therein). These studies suggested that the interference with intracellular signaling pathways resulted from cis-associations with other cell surface receptors and the presence of the conserved Tyr320 and Cys335 residues in their cytoplasmic domain. The phosphorylation status of these amino acids is known to regulate membrane movement and allow physical co-localization with cell surface receptors, thus interfering with intracellular signaling pathways. Evidence that this occurs with several co-receptors, adhesion molecules, Toll-like receptors, and cytokine receptors [188,189,190,191]. This is an area warranting further investigations.

## 6. Open MHC-I Conformers: Unforeseen Modulators of Neuronal Development and Synaptic Plasticity?

### 6.1. Current Knowledge

Contrary to what was assumed for many years, neurons and glial cells express MHC-I molecules, playing crucial physiological roles under healthy conditions. Following the serendipitous discovery that MHC-I expression was required for central nervous system (CNS) development [192,193], many studies conducted in genetically modified mouse models have attempted to dissect and understand the molecular cues whereby expression of MHC-I molecules in the CNS regulate essential neurobiological processes. Indeed, these groundbreaking studies opened new avenues of research and pointed to MHC-I molecules as crucial regulators of brain organogenesis and factors for the development of neurological disorders, two challenges foreseen more than 40 years ago [16,17]. At present, MHC-I molecules have been detected in cells of the CNS and the peripheral nervous system (PNS) of several animal species, including humans, playing critical roles in neurogenesis, synaptic plasticity and neuronal communication [194,195,196,197,198]. Indeed, the aforesaid original studies were conducted in neurons undergoing activity-dependent synaptic remodeling and revealed that metabolically active neurons drove the reshaping. In this respect, elegant in vitro studies using cultures of embryonic mouse retina explants within a short distance from thalamic explants obtained from wild-type mice or NSE-H-2D^b^ transgenic mice (i.e., mice whose neurons express high levels of H-2D^b^ molecules) provided insightful results [199,200]. Thus, retina neurites outgrew to form connections with wild-type but not with NSE-H-2D^b^ thalamic explants due to growth inhibition. Notably, the inhibitory effect observed with NSE-H-2D^b^ thalami explants was mediated by soluble H-2D^b^ conformers. Additionally, the studies showed that cis-interactions of open H-2D^b^ conformers with surface receptors promoted neurogenesis. In contrast, trans-interactions of closed H-2D^b^ conformers with cell surface receptors caused inhibition of neurite growth. These data are enlightening in the sense that they show, for the first time, a role for open MHC-I conformers in regulating neurogenesis. They are reminiscent of the results obtained with antibodies against open HLA-I conformers and provide clues about the possible mechanisms used by MHC-I molecules to regulate CNS architecture and functioning.

### 6.2. Forward-Looking Perspective

In the context of the current knowledge described in the previous sections, it is conceivable to think that the physiological equilibrium that exists between closed and open conformers at the plasma membrane of metabolically active cells, and the resulting formation and shedding of open conformers, will also take place in cells of the CNS. Thus, in line with what was described in the previous sections, cell surface MHC-I molecules in the CNS and PNS could have three fates.

First, MHC-I molecules may cis-associate or trans-interact with inhibitory receptors such as PirB (in mice) and LIRLB (in humans). Indeed, several studies have provided evidence that cis–trans-interactions between MHC-I molecules and inhibitory receptors inhibit axonal growth and regeneration [201,202], which agrees with the inhibitory effect of these cis-interactions in NK cytotoxicity [109]. Second, MHC-I molecules may cis-associate with growth factor receptors, such as the IR, and modulate IR-mediated signaling. Indeed, ligation of insulin to its receptor regulates synapse number and dendritic plasticity in vivo [133]. In that regard, a recent study has shown that MHC-I molecules inhibit neuronal IR-mediated signaling, thus limiting hippocampal synapse density [134]. Whether the influence of MHC-I molecules on IR-signaling results from cis-associations, trans-interactions, interactions mediated by soluble conformers, or part of all of them, remains a critical issue that needs to be answered. Indeed, the inclusion of antibodies recognizing open MHC-I conformers in these studies will likely provide insightful results. Third, MHC-I molecules may be shed as soluble conformers (both closed and open) and regulate neurogenesis and neuronal polarization [199,200].

Altogether, these studies illustrate the complexity that surrounds the role of MHC-I molecules on CNS physiology and how a deviation on the baseline levels of expression of MHC-I molecules caused by inflammatory or anti-inflammatory conditions may impact brain function and cognition. In this respect, a recent study in the mouse CNS has unveiled cryptic motifs (PDZ sequences) in the cytoplasmic tail of MHC-I molecules [203]. These PDZ sequences regulate protein localization and the formation of scaffolds involved in cell–cell contact at immunological and neuronal synapses [204,205]. Notably, these PDZ motifs contain the conserved Tyr320 and Ser335 residues, targets of in vivo phosphorylation/de-phosphorylation, post-translational modifications that identify cell surface open and closed conformers [5,8,9,10,11,12,13,14,15].

## 7. Concluding Remarks and Future Prospects

Currently, there is no doubt that the exquisite molecular and functional features held by the structure of the αHC are shared by all MHC-I molecules expressed by every nucleated cell and platelet. Thus, cis–trans interactions with immunoreceptors and receptors for hormones, growth factors, and other signaling factors have important physiological implications for cell growth, proliferation, survival, and differentiation of normal and diseased cells. Additionally, open sHLA-I conformers’ shedding has physiological consequences for cell–cell communication, either directly or through the generation of anti-sHLA-I antibodies. The studies summarized and analyzed in this review have attempted to integrate all the published data on open MHC-I conformers. It will not be difficult for a thoughtful reader to find connections and similarities between findings reported in the 1980s/1990s and findings reported in the last decade, with the IR being a common nexus. The main goal of this review is to stimulate and direct further research on this crucial topic to elucidate the molecular cues behind the known associations between human HLA-I alleles and the development and progression of chronic inflammatory disorders, including neurodegenerative disorders. Among these, the metabolic disorder type 2 diabetes deserves special mention.

Thus, the early studies unveiling the physiological relevance of the cis-association between open HLA-I conformers and the IR:insulin complex, namely the regulation of the activity of intracellular kinases PI3K, ERK, and AKT, are inextricably linked to recent studies that have deepened our understanding of the physiological importance of modulating intracellular metabolic pathways in cancer biology and CNS function [206]. The study of the effect of the expression of open HLA-I conformers in these two areas of knowledge is relatively uncharted territory. However, the existence of close connections between HLA-I molecules and IR-mediated signaling [51,134], progression of cancer [83,84,85,86,87,122,123,124,125,126] and development of cognitive disorders [207,208,209,210,211,212] is substantial grounds to keep clearing the way towards a better understanding of the original function of MHC-I molecules [6,7,8,9].

## Figures and Tables

**Table 1:** Some reported cis-associations between MHC-I molecules and cell surface receptors *.

Year	Main Findings	Involved Alleles	Ref.
1983	Physical association between H-2 class I molecules and insulin receptors in mouse liver membranes	H2-K^b^, H-2D^b^	[90]
1984	Physical association between HLA-I molecules and epidermal growth factor (EGF) receptors in cancer cells and fibroblasts	Not determined	[96]
1988	Physical association between HLA-I molecules and CD8 receptors on activated normal human T cells	Not determined	[105]
1988	Binding of luteinizing hormone to its receptor triggers an association with H-2 class I molecules in Leydig cells	H-2D^d^, H-2D^k^, H-2K^d^, H-2K^k^	[97,98]
1988/1990	Physical association between HLA-I molecules and the IL-2R α and β chains in normal and transformed T cells	Not determined	[101,102]
1990	Functional interaction between H-2 class I molecules and β-adrenoceptors in cardiac membrane preparations	H-2D^k^, H-2K^k^	[99]
1991	Interactions between HLA-I molecules and γ-endorphin receptors on activated T cells and transformed B cells	Not determined	[100]
1991	Physical association between H-2 class I molecules and CD8αβ receptors on T cells	H-2K^k^	[106]
1993	Physical association association of insulin receptors with four different class I human leukocyte antigen molecules on cell surfaces	HLA-A1, HLA-A2, HLA-B5, and HLA-B8	[112]
1995	Physical association between HLA-I molecules and the transferrin receptor in B lymphoblastoid cell lines	Not determined	[104]
1997	Physical association between HLA-I molecules and the tetraspanin protein CD82 in human B cell lines	HLA-A2, HLA-A23, HLA-B5, HLA-B8, HLA-B13	[107]
1998	Physical interaction between H-2 class I molecules and the N-terminal domains of the CD8α and CD8β receptors	H-2L^d^	[48]
2004	Clusters containing HLA-I molecules and α, β, and ɣc chains of the IL-2/IL-15 receptors in a leukemia T cell line	Not determined	[64]
2004	Physical association between H-2 class I molecules and Ly49 receptors on NK cell transfectants	H-2D^d^, H-2D^k^	[109]
2004	Physical association between HLA-I molecules and CD8αβ-Lck in normal human T cells activated in vitro	Not determined	[14]
2006	Physical interaction between HLA-B27 molecules lacking Tyr320 and transferrin receptors in a thymoma cell line	HLA-B27	[15]
2007	Physical association between HLA-I molecules and LILRB2, and its mouse ortholog PirB, in mast cells	Not determined	[110]
2008	Physical association between HLA-I molecules and LILRB receptors, and their mouse ortholog PirB, in osteoclasts	Not determined	[111]
2010	Physical association between HLA-I molecules and the β4 integrin in transfected endothelial cells	HLA-A2, HLA-B56	[113]

* For a complete listing see [5].

## Data Availability

Not applicable.
